# Sensitivity and concordance study of an open source EPID‐based linear accelerator test suite

**DOI:** 10.1002/acm2.70569

**Published:** 2026-04-14

**Authors:** Lana C. Critchfield, Cory S. Knill, Ellie Durussel, Justin K. Mikell, Michael Barnes, Michael Pillainayagam, William P. Donahue, Seng Boh Lim, John J. DeMarco, Mario Perez, Nilendu Gupta, Hania Al‐Hallaq, Richard A. Popple, Jim Irrer, Jean M. Moran

**Affiliations:** ^1^ Department of Radiation Oncology University of Michigan Wyoming Michigan USA; ^2^ Department of Radiation Oncology University of Michigan Ann Arbor Michigan USA; ^3^ Department of Radiation Oncology University of Michigan Essexville Michigan USA; ^4^ Department of Radiation Oncology Washington University St. Louis Missouri USA; ^5^ Department of Radiation Oncology Calvary Mater Hospital Newcastle NSW Australia; ^6^ Memorial Sloan Kettering Cancer Center Samford Connecticut USA; ^7^ Radiation Dosimetry Core Memorial Sloan‐Kettering Cancer Center New York New York USA; ^8^ Department of Radiation Oncology Cedars‐Sinai Medical Center Los Angeles California USA; ^9^ Northern Sydney LHD Sydney NSW Australia; ^10^ Department of Radiation Oncology The Ohio State University, James Cancer Hospital Columbus Ohio USA; ^11^ Department of Radiation Oncology Emory University Atlanta Georgia USA; ^12^ University of Alabama Birmingham Birmingham Alabama USA; ^13^ Memorial Sloan Kettering Cancer Center New York New York USA

**Keywords:** EPID, linear accelerator, quality assurance

## Abstract

**Background:**

Electronic portal imaging devices (EPIDs) enhance linear accelerator (linac) quality assurance (QA) efficiency but are not universally used beyond patient‐specific IMRT QA. A multi‐institutional Consortium seeks to standardize linac QA utilizing an EPID‐based test suite.

**Purpose:**

This study evaluates the sensitivity of a test suite to intentional linac and plan errors and the concordance with linac fluctuations over time.

**Methods:**

Baseline and error‐introduced tests were performed on a Varian TrueBeam and Clinac. Evaluated parameters included enhanced dynamic wedge factors (EDW) (6MV), central axis dose (6MV, 6MV FFF, 16MV), focal spot alignment and beam symmetry (6MV, 16MV), dose rate‐gantry speed and multi‐leaf collimator (MLC) leaf speed (6MV). Errors included varying EDW angles (10°–60°), output changes, beam steering for focal spot and symmetry inaccuracies, and meterset adjustments of control points for dose rate‐gantry speed and MLC leaf speed tests. Measurements were performed with an EPID, analyzed via test suite software, and compared to an ion chamber (IC) or array. Concordance analysis was performed on a single linac over 5 months to assess the test suite's ability to quantify deviations during routine operations. Measurement reproducibility was assessed.

**Results:**

Results were reported as differences between EPID and an IC or array. For each EDW, wedge factor differences were <0.5% (TrueBeam) and <1.5% (Clinac). Central axis dose variations stayed within ±0.8%. For focal spot, EPID underestimated the radial direction and overestimated the transverse direction. Symmetry measurements showed strong linearity (*r* > 0.99), though EPID measurements underestimated changes in symmetry relative to baseline. Maximum differences for dose rate‐gantry speed and MLC leaf speed were <0.4%. In the concordance study, output differences averaged 0.3% ± 0.3% (6MV), 0.5% ± 0.3% (6MV FFF), and 0.3% ± 0.2% (16MV). Systematic differences in symmetry were observed between EPID and a 2D‐array with standard deviations <0.2%.

**Conclusions:**

Comparisons with traditional detectors showed the EPID test suite can detect errors and supports commissioning and clinical integration.

## 1 INTRODUCTION

Radiation therapy treatments, utilizing MV photon beam external beam therapy, represent a sophisticated approach involving the application of high‐energy radiation directed at patients to treat cancer and other ailments. Over the past few decades, substantial technological advancements have been made to enhance the precision of radiation delivery. These advancements encompass sophisticated delivery, imaging, and computational techniques. The effectiveness of these enhanced technologies depends on their accuracy, necessitating rigorous quality assurance (QA) measures to ensure optimal performance. Organizations like the American Association of Physicists in Medicine (AAPM), along with other scientific and professional bodies, have developed reports to guide physicists in establishing QA protocols.^1^, ^2^, ^3^ The growing intricacy and volume of QA tasks have significantly increased the workload for clinical medical physicists, posing challenges in task completion.^4^, ^5^ Therefore, it is crucial to persist in developing more efficient QA techniques and processes to alleviate the mounting burden on clinical physicists.

Linear accelerator (linac) QA efficiency improvements have been achieved through the advancement of novel measuring devices, tests, and analytical techniques. One notable improvement involves utilizing the electronic portal imager (electronic portal imaging device [EPID]) for QA purposes.^6^, ^7^ Originally designed to replace traditional film in capturing localization images and visualizing field apertures on patient anatomy, EPIDs were initially employed for QA tasks traditionally carried out using film, including the Winston–Lutz test and picket fence test.^8^, ^9^, ^10^, ^11^ These initial applications have expanded to encompass EPID‐based QA for multi‐leaf collimator (MLC) and jaw calibrations, patient‐specific measurements, as well as outputs, profiles, and isocentricity quantification.^12^, ^13^, ^14^, ^15^, ^16^, ^17^, ^18^, ^19^

Recognizing its extensive utility and widespread availability, both linac and QA vendors, along with independent physicists, have developed EPID‐based tests and associated analysis software.^20^, ^21^, ^22^, ^23^ Furthermore, AAPM Task Group Report 332^24^ and IPEM topical report: guidance for the use of linac manufacturer integrated quality control^25^ provide extensive guidance on independent evaluation of such systems, which are typically considered to be a “black box”. In contrast to vendor‐supplied QA tests and analysis is Automated Quality Assurance (AQA), a multi‐institutional consortium, which aims to “streamline automation and provide a common data repository that can be leveraged for operational benefits and research opportunities.” To fulfill this objective, AQA uses a team science approach to developing, testing, and validating linac QA and has created a “white‐box” web‐based platform for QA publicly available through an open‐source code (https://github.com/irrer/aqa), featuring multiple tests utilizing EPID capabilities.^23^ The standardized test suite's easy accessibility makes it a candidate for routine QA, establishing baselines for credentialing, cross‐institutional comparisons, cost‐effective solutions for developing nations, backup for routine QA, independent verification of vendor‐supplied solutions, and utilization in research investigations. A hybrid QA solution was well demonstrated by Pearson et al., in which the authors developed and validated a program with both automated commercially available elements, such as Varian's MPC, as well as conventional approaches utilized at a reduced frequency.^26^ This test suite could similarly prove useful in building a unique hybrid QA program.

Although the test suite and other commercial EPID QA software packages have proven valuable, a comprehensive evaluation of their testing methods and analysis is imperative before widespread adoption. Initial evaluations, which are generally not tied to specific software packages, have focused on assessing the performance of individual measurement techniques. These evaluations are often conducted by the tests’ inventors and subsequently validated by other medical physicists. However, as new implementations of these tests are introduced that involve software for analysis, thorough commissioning becomes necessary, as the software's approach to analyzing results can significantly impact the accuracy and precision of the test itself. Furthermore, EPID‐based tests are still often relative measurements, where results are compared to baselines established during the initial commissioning of the test. Subsequently, the test is used to measure deviations from these baselines for routine QA. Considering this, the goal of this study was to assess the sensitivity of selected test suite analyses in detecting deviations introduced into clinical EPID and linac systems. This initial technical validation was performed at a single consortium institution and included two linacs. The findings can inform the evaluation of other EPID‐based QA analysis software packages, potentially leveraging the consortium test suite as a tool for commissioning vendor‐supplied QA test suites.

## 2 METHODS

The validation of the AQA test suite's ability to detect deviations was performed in two distinct phases:

1. Sensitivity studies—An initial validation where deviations of varying magnitudes were intentionally introduced into the linacs. The test suite's ability to detect these deviations was then evaluated.
2. Concordance studies—A multi‐month analysis assessing the test suite's ability to quantify deviations that naturally occur during the routine operation of the linacs.

In both phases, deviations measured with the test suite EPID analysis were compared to an external gold standard, using ion chamber (IC) measurement obtained with either an A12 IC or an IC array. The specific methods used for each individual test are detailed in the following sections.

### 2.1 Sensitivity analysis

The sensitivity analysis focused on tests including enhanced dynamic wedge factors (EDW), central axis dose (output), symmetry, focal spot, MLC leaf speed, and dose rate versus gantry speed. Dark and flood field calibrations were performed on all EPIDs before the first measurement of each test and remained unchanged throughout image acquisition. Dark field calibration will have a small effect on central axis dose measurements. Flood field correction will have a resetting effect on symmetry. The Calibrated Unit (CU) calibration for Portal Dosimetry (Varian Medical Systems) had been performed, which will affect the output results, but remained unchanged throughout the image acquisition. Measurements were conducted using either a Varian Clinac 2100 equipped with an AS500 EPID or a Varian TrueBeam equipped with an AS1200 EPID.^27^ All EPID images were acquired with the panel positioned at 150 cm source to imager distance (SID), with no obstructions in the beam path. Images were acquired using Integrated Image mode. Three measurements were used per data point for EPID, IC, and IC array results.

Reproducibility was assessed for all measurement devices by acquiring five consecutive measurements without altering the setup and calculating the standard deviation of the results. These reproducibility results were then propagated through calculations using standard error propagation techniques. Uncertainties are represented in subsequent figures as error bars.

#### 2.1.1 EDW

Wedge factors (WFs) were measured on both the Clinac and a TrueBeam using EPIDs and IC Profiler (ICP) (Sun Nuclear Corp, Melbourne, Florida). ICP measurements were performed at 150 cm sourced to surface distance (SSD) with 5 cm of solid water for backscatter and no additional buildup.

The EPID analysis selects a 5 mm wide region of pixels in the center of the image for analysis, approximately 22 pixels. The ability of the test suite EPID analysis to detect deviations in WFs was assessed by adjusting the delivered wedge angle between 10° and 60° on the console and comparing the EPID‐measured WF to the ICP‐derived values.

#### 2.1.2 Central axis

Central axis dose outputs were measured on both the Clinac and TrueBeam using EPIDs and IC. IC measurements were performed in solid water at a depth of 10 cm with a 90 cm SSD and an 18 × 18 cm^2^ field size. Due to EPID energy restrictions on the Clinac, only a 6MV beam was used. However, on the TrueBeam, output measurements were conducted for 6MV, 6MV flattening filer free (FFF), and 16MV beams. The average CU of the pixels within a central 5 mm radius circle is used for EPID analysis, approximately 1564 pixels. The region of interest (ROI) variable is configurable within the test suite and can be changed as needed to better reflect the size of the specific IC being used in comparison. The ability of the test suite EPID analysis to detect dose deviations from baseline was assessed by adjusting the delivered monitor units (MU) between 50 and 60 on the console and comparing the EPID‐measured output to the IC‐measured values.

#### 2.1.3 Focal spot

Focal spot measurements were performed on a non‐clinical Clinac using an EPID and ICP. A non‐clinical machine was used because a focal spot misalignment could result in the beam colliding with components in the beam line, causing costly damage and repairs which would be time consuming for a clinical machine. EPID results were compared to ICP measurements taken in a gantry‐mounted setup with 1 cm of additional solid water (1.9 cm total buildup).

The EPID‐based focal spot is based upon the method of Chojnowski et al. and the test requires four fields: two with opposing collimator angles defined by the MLCs and two with opposing collimator angles defined by the jaws.^28^ This test analyzes EPID images using edge detection and linac geometry to account for the MLCs and jaws being at different heights within the collimator head. The result is the total deviation from the central axis.

The ICP method was first presented in Barnes and Greer.^29^ The ICP method requires two fields: one measured at a collimator angle of 90° and another at 270°. The focal spot position is then determined using the following equation:

$$\mathrm{Focal}\;\mathrm{spot}=\frac{-\left(CA_{90}-CA_{270}\right)}{2}$$

where CA_90_ and CA_270_ represent the offsets from the central axis for beam profiles taken at collimator angles of 90° and 270°, respectively. This calculation is repeated for both the *x* and *y* directions.

The ability of the test suite EPID analysis to detect deviations in focal spot position was evaluated by adjusting the radial and transverse positioning servos to shift the focal spot up to 0.6 mm from baseline near the central axis. Beyond this magnitude, the dose‐rate decreased substantially making measurement difficult. After each adjustment, the angle servos were modified to produce a symmetric beam, simulating a clinical scenario where the focal spot is misaligned while maintaining beam symmetry. EPID‐measured focal spot deviations were then compared to ICP‐derived values.

#### 2.1.4 Symmetry

Symmetry measurements were performed only on a decommissioned Clinac using an EPID and ICP, due to the same reluctance to adjust a clinical beam as with focal spot testing. For the EPID‐based symmetry test, an 18×18 cm^2^ field was delivered. The test suite software calculates symmetry on flood field calibrated images by sampling five 5 mm circular regions (∼1500 pixels): one at the central axis and four positioned 75 mm away (approximately 83.33% of the field width) in the four cardinal directions. Symmetry is determined using the following equations:

$$\mathrm{axial}\;=\left(\frac{\mathrm{top}-\mathrm{bottom}}{\mathrm{bottom}}\right)\;*100$$

$$\mathrm{transverse}\;=\left(\frac{\mathrm{right}-\mathrm{left}}{\mathrm{left}}\right)\;*100$$

These results were compared to measurements taken with an ICP placed on the treatment couch, with 5 cm of backscatter and solid water buildup to the depth of dose maximum, utilizing the same definition of symmetry as above.

The ability of the test suite EPID analysis to detect symmetry deviations was evaluated by adjusting the radial and transverse angle servos to create beams with up to 10% asymmetry. Larger deviations reduced dose rate, limiting measurements. EPID symmetry values were compared with ICP‐measured values. Since EPID symmetry measurements are relative to baseline and are not absolute, the initial pre‐steering measurements were subtracted from subsequent measurements for both EPID and ICP results. Thus, the analysis assessed the EPID's sensitivity to changes in symmetry, not its ability to measure absolute symmetry.

#### 2.1.5 MLC tests

Tests involving MLC motion included volumetric modulated arc therapy (VMAT) dose rate‐gantry speed and VMAT MLC leaf speed assessments, tests originally created by Ling et al. using film.^30^ Fredh et al transferred these tests onto an EPID and analyzed using an in‐house software.^31^ These tests are available at the Varian webpage (www.myVarian.com). Dose rate‐gantry speed and MLC leaf speed were conducted on both the Clinac and TrueBeam using EPIDs and an ICP. EPID results were compared to ICP measurements taken in a gantry‐mounted setup with 1 cm of additional solid water (1.9 cm total buildup). Detector gantry mount sag was not measured and considered negligible based on the ROI positions set in from the field edge.

The ability of the test suite EPID analysis to detect deviations in MLC performance was evaluated by systematically modifying the baseline test conditions using an in‐house MATLAB script to alter the DICOM files. The baseline dose rate‐gantry speed test involved delivering the same MU for both an open static field and a VMAT dynamic field with seven equally spaced segments, each delivering a set MU with varying dose rates. The baseline MLC leaf speed test consisted of delivering a set MU for both an open static field and a VMAT dynamic field with four equally spaced MLC sliding window segments, each delivering the same MU at different dose rates and leaf speeds. The baseline tests are analyzed by first normalizing to an open field image. Then, the mean from a large ROI (1 cm × 16 cm for MLC leaf speed and 0.75 cm × 17 cm for dose rate‐gantry speed) is recorded for each segment. The mean is averaged between strips and then each strip is compared to the average with a 2% tolerance. Deviations were introduced by modifying the cumulative meterset weight of the control points in the DICOM files. For the modified dose‐rate gantry speed test, the seven individual segments were adjusted to each deliver different amounts (between 8% and 16%) of the total MU while maintaining the varying dose rates of the baseline test. For the modified MLC leaf speed test, the four segments were adjusted to deliver 16%, 21%, 29%, and 34% of the total MU, respectively. The open static field parameters are the same for both baseline and modified tests.

Results for both tests were determined by analyzing output measurements of the segments. For the MLC leaf speed test, the ROI within each band is 1 cm × 16 cm (∼33 000 pixels). For the dose rate‐gantry speed test, the ROI within each band is 0.75 cm × 17 cm (∼25 000 pixels). The ROIs did not extend beyond the field edge; however, it is possible that the edge of an ROI intersected individual pixels within the field. In such cases, the fractional pixel value within the ROI was used. The calculations for both tests followed the same approach. For each band within the seven segments, the center *R*_LS_ represents the average calibration units (CU) or output for the dynamic field, while *R*_open_ represents the average CU or output for the open field. Using these values, the following metrics were calculated:

$$\;R_{\mathrm{corr}\left(x\right)}=\left(100*R_{\mathrm{LS}\left(x\right)}\right)/R_{\mathrm{open}}$$

$$\mathrm{Diff}\;\left(x\right)=R_{\mathrm{corr}\left(x\right)}\;-\mathrm{Average}\left(R_{\mathrm{corr}}\right)$$

**FIGURE 1:**
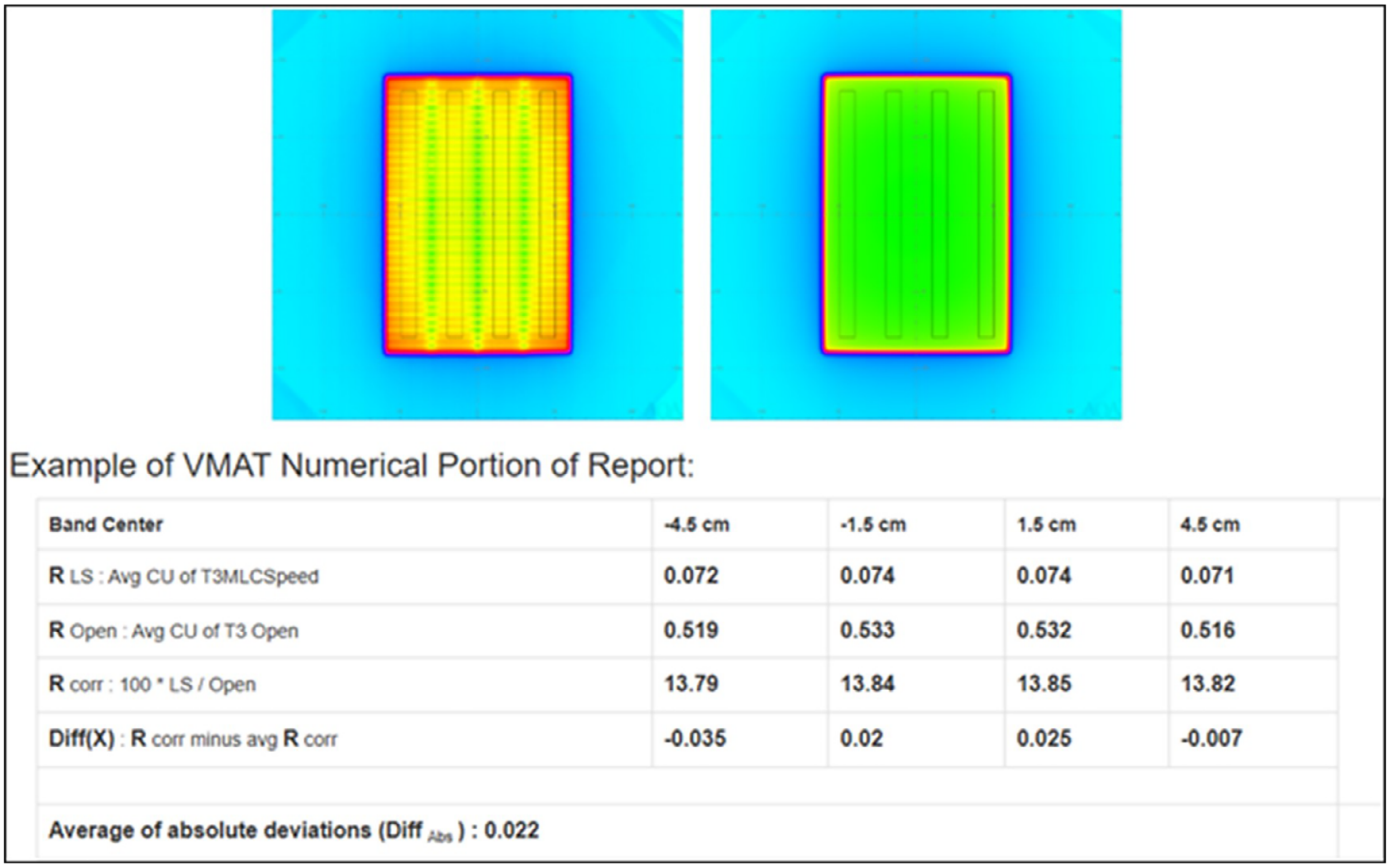
The figure above shows an example VMAT MLC leaf speed test and results as displayed on the consortium website (https://automatedqualityassurance.org/static/doc/VMAT/index.html). MLC, multi‐leaf collimator; VMAT, volumetric modulated arc therapy.

Figure 1 displays an example VMAT MLC leaf speed test and results. The Diff(*x*) values were calculated for each band segment in both the baseline and modified plans. EPID‐measured results were then compared to ICP‐derived values along the central axis (*y* = 0) at the corresponding position with each detector system. A similar comparison was done for the dose rate‐gantry speed test (detectors positioned at *x* = 5.1, −3.1, −1.1, 0.9, 2.9, 4.9, 6.9 cm) and MLC leaf speed test (detectors at position *x* = −4.5, −1.5, 1.5, 4.5 cm), both tests utilizing one detector per segment position.

### 2.2 Concordance analysis

**FIGURE 2:**
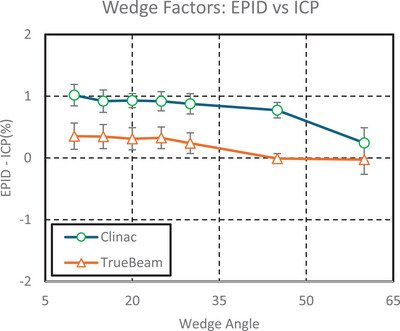
Comparison of the percent difference between wedge factors measured with an ICP and the test suite EPID analysis on a Clinac and a TrueBeam. EPID, electronic portal imaging device; ICP, IC Profiler.

Concordance analysis was conducted over a 5‐month period (*n* = 5), during which beam output, flatness, and symmetry were measured monthly using the test suite EPID‐based QA tests and compared to an IC and QABC (daily QA IC array) measurements. All measurements were acquired on a single TrueBeam linac equipped with an aS1200 imager. Output and symmetry measurements were evaluated relative to baseline (first month of measurement) to monitor drift between the two methods over time. A 5‐month observation period was chosen as sufficient to detect potential drift or changes to machine performance while remaining short enough not to impede clinical operations.^25^ This timeframe ensures an adequate dataset for identifying meaningful trends Figure 2.

The test suite EPID measurements were performed with the imager at an SID of 150 cm, consistent with both commissioning and clinical setups. IC measurements were conducted in a solid water configuration, replicating the clinic's monthly QA acquisition, while QABC measurements mirrored the clinic's daily QA calibrations. This approach allowed the concordance analysis to assess the test suite's potential for integration into both monthly and daily QA workflows.

## 3 RESULTS

### 3.1 Sensitivity analysis

#### 3.1.1 EDW

The percent difference between ICP and the test suite EPID measurements for WFs was less than 0.4% for the TrueBeam and less than 1.1% for the Clinac. The ICP demonstrated a WF reproducibility uncertainty of 0.1%, and the test suite analysis demonstrated a reproducibility uncertainty of <0.1% Figure 2.

#### 3.1.2 Central axis dose

**FIGURE 3:**
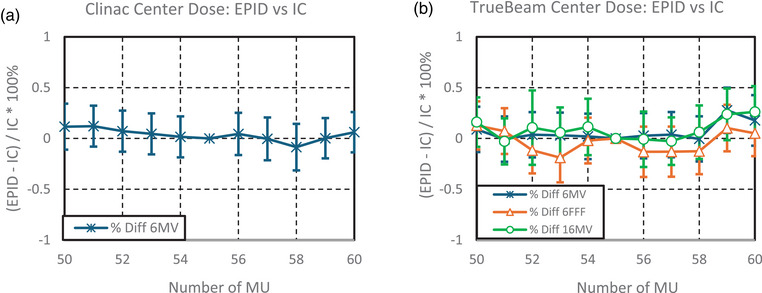
(a, b) The percent difference between IC measurements and the test suite EPID results for central axis dose. EPID, electronic portal imaging device; IC, ion chamber.

The percent differences between the test suite EPID and IC data varied across beam configurations. For the TrueBeam 6MV, 16MV, and 6MV FFF beams, differences were within ±0.8%, with fluctuations across the MU range. The 6MV Clinac beam exhibited similar variation, with differences also staying within ±0.8%. Data for both the Clinac and TrueBeam are displayed in Figure 3a and b, respectively. Reproducibility uncertainty in the test suite central dose analysis was 0.025 cGy, accounting for approximately 0.04% of the delivered dose. In IC measurements, the reproducibility uncertainty of the IC, electrometer, and linac combination was 0.002 nC, corresponding to about 0.02% of the delivered dose.

#### 3.1.3 Focal spot

**FIGURE 4:**
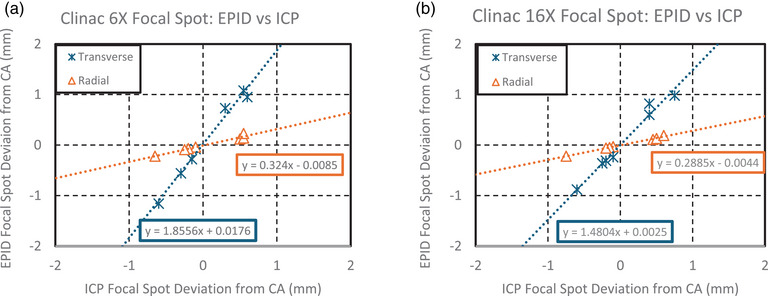
(a, b) The above figures show the transverse and radial focal spot deviation from central axis (CA) during beam steering.

Compared to the ICP, the test suite EPID method underestimated measurements in the radial direction and overestimated them in the transverse direction, as shown in Figure 4a and b, for both 6MV and 16MV beams. A linear relationship was observed between the two methods. The ICP demonstrated a reproducibility uncertainty of <0.03 mm, while EPID reproducibility was <0.07 mm.

#### 3.1.4 Symmetry

**FIGURE 5:**
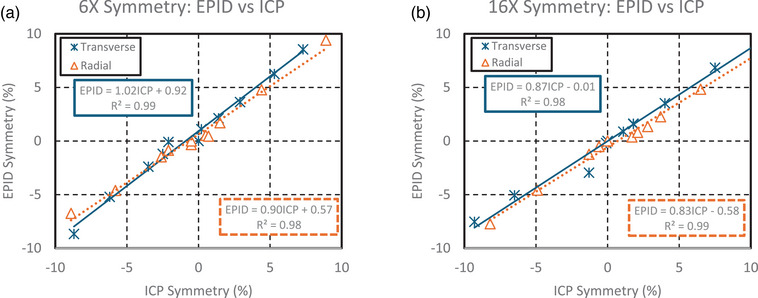
(a, b) The graphs above show the transverse and radial symmetry for the ICP (*x*‐axis) compared to the EPID results (*y*‐axis). EPID, electronic portal imaging device; ICP, IC Profiler.

A linear relationship was observed for symmetry measurements between the ICP and the EPID, as shown in Figure 5a for the 6MV beam and Figure 5b for the 16MV beam. For both energies, the transverse symmetry slope was closer to the ideal value of one compared to the radial direction. The largest deviation was a slope of 0.83 in the radial direction of the 16MV beam, meaning that when the ICP measured a 2% change in symmetry, the EPID underestimated the change, reporting a 1.66% change from baseline. The ICP demonstrated a reproducibility uncertainty of <0.05% for symmetry in both the radial and transverse directions, while EPID reproducibility was 0.08% in both directions.

#### 3.1.5 MLC

**FIGURE 6:**
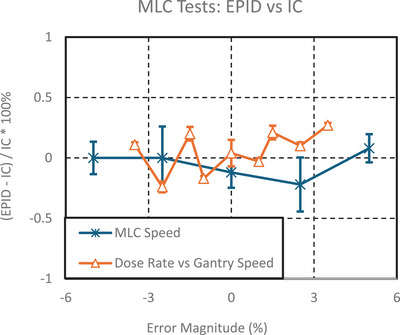
The graph above shows the difference between the ICP method and the test suite EPID method for both MLC‐based tests in relation to the magnitude of error. EPID, electronic portal imaging device; ICP, IC Profiler; MLC, multi‐leaf collimator.

The maximum difference in Diff(*x*) values between the ICP and EPID for the dose rate‐gantry speed test was 0.3% for the baseline and 0.3% for the modified tests, as shown in Figure 6. For the MLC leaf speed test, the maximum difference in Diff(*x*) values was 0.4% for the baseline and 0.3% for the modified tests.

For dose rate‐gantry speed, the reproducibility uncertainty in the test suite analysis for each position was, on average, 0.01% for both the EPID and ICP methods. For MLC leaf speed, the test suite analysis showed an average reproducibility uncertainty of 0.09% for the EPID and 0.08% for the ICP method.

### 3.2 Concordance analysis

**FIGURE 7:**
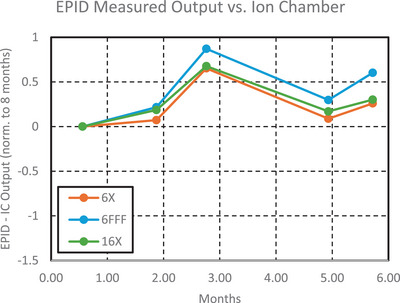
Difference in beam output measured by the test suite electronic portal imaging device (EPID) and QABC ion chamber array over 5 months, normalized to the first month.

**FIGURE 8:**
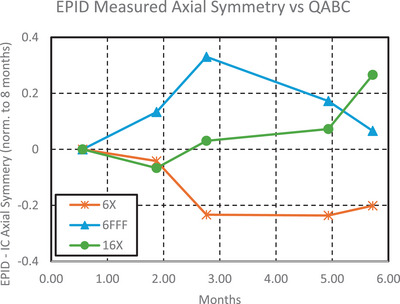
Difference in radial symmetry measured by the test suite electronic portal imaging device (EPID) and QABC ion chamber array over 5 months, normalized to the first month.

**FIGURE 9:**
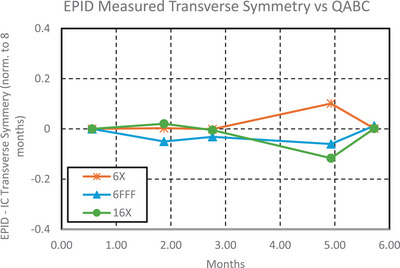
Difference in transverse symmetry measured by the test suite electronic portal imaging device (EPID) and QABC ion chamber array over 5 months, normalized to the first month.

Output differences between the test suite and IC were on average 0.3% ± 0.3%, 0.5% ± 0.3%, and 0.3% ± 0.2% for 6MV, 6MV FFF, and 16MV, respectively, as can be seen in Figure 7. There were systematic differences between the test suite and QABC in radial symmetry, transverse symmetry, but the standard deviations were < 0.2%, as can be seen in Figures 8 and 9.

## 4 DISCUSSION

The AAPM Medical Physics Practice Guideline 8.b underscores the importance of integrating automation in machine QA programs with a comprehensive understanding of the QA techniques and analysis employed.^3^ Achieving this requires access to the programming code used for calculations, yet proprietary restrictions often limit this access in vendor‐supplied automation. Therefore, our study presents a methodology for commissioning automated linac QA methods by evaluating both concordance and sensitivity. These methods could prove useful when detailed knowledge of the methodology used for QA calculations is unavailable. This method aligns with TG‐332′s recommendations for commissioning a “black box”, where the inner calculations are inaccessible, by benchmarking against a ground truth and performing sensitivity and robustness checks. Essential to this commissioning process is comparing the automated QA method to a known and trusted standard. In our study, this standard was an ICP and an IC; however, alternate detectors or analysis systems that have been commissioned properly and deemed suitable could be equally effective. The methods explored above are not novel to this study, for example, Greer and Barnes investigated the use of an EPID for measurement of EDWs.^32^ They demonstrated agreement between an EPID and diode array to within 2% for all WFs. This corresponds with the findings above, supporting the use of the test suite for EDW measurements and bolstering the significance of comparing EPID results to a 2D‐array as a trusted standard. For the sensitivity analysis of WFs, it is important to note that the difference of 1.1% observed between the Clinac EPID and ICP is substantial, given the recommended QA tolerance of 2%.^1^ The Clinac utilizes an older EPID system compared to the TrueBeam, which could account for these discrepancies. In addition, the maximum absolute difference between the Clinac EPID and ICP was 1.1%, occurring at a 10° wedge angle; this difference decreased to 0.8% at a 30° wedge angle. This suggests a consistent offset in the EPID‐measured wedge angle. If a relative comparison were performed, similar to the other EPID tests, this consistent offset would be present in both the initial baseline and routine QA measurements and would therefore have a smaller impact on detecting relative changes from baseline. Consequently, this test could serve as a preliminary verification that a machine is suitable for clinical use. It would be expected that a warning or failure on the test suite measured EDW relative to baseline would warrant the use of an ICP as the gold standard for precise verification. In the range of clinically acceptable beam symmetry (within ±2%), the EPID and ICP agreed within 1.0% for 6MV, similar to the findings of Sun et al. which demonstrated agreement within ±1.2%.^22^ Similarly, Sun et al. employed the use of beam steering in order to deliberately create asymmetric beams with validation using a 2D IC array. Although that study utilized a phantom, the similar method of test validation has proven effective in both studies.

After the sensitivity analysis, a concordance assessment should begin.^24^, ^25^ Using the same methods and devices from the sensitivity phase can aid in comparisons, but daily QA devices may provide more frequent data collection opportunities. Alternatively, a hybrid approach can be adopted where daily QA devices are used for consistent data gathering, and the commissioning devices are periodically employed to maintain a connection between commissioning and ongoing analysis.^26^ Daily QA devices offer immediate feedback and can help identify potential inaccuracies for investigation, such as hardware changes or errors in test execution by staff. Although less frequent and more time‐consuming than daily QA, comparisons with the more accurate devices used in the sensitivity phase will ensure the test provides a reliable assessment of machine performance and will provide accurate data for setting action limits, reducing the risk of false positives from the test limitations.

Whenever possible, commissioning and concordance data should be reviewed for potential practical improvements in data acquisition and test execution. For instance, during output verification reproducibility measurements, it was observed that the output on the setup field, used to determine the collimator center with the EPID data, drifted by 0.1%–0.2%, likely due to EPID ghosting effects from previous measurements.^33^ Although this level of uncertainty may be acceptable for routine daily output verification, it is important to consider that a 0.2% uncertainty might arise if the EPID is calibrated after prolonged use, while typical daily QA measurements are performed in the morning, before any prior irradiations to the EPID. Given the additional uncertainty, the action level should be set more conservatively than for a device with higher accuracy. By combining a tighter tolerance with a more uncertain method, it becomes more likely that genuine failures will be detected, as the stricter threshold helps ensure that potential problems are not overlooked due to the method's inherent variability. The stability of the EPID should be carefully evaluated for ongoing QA. Both dose response constancy and relative response constancy are important considerations. Dose response constancy, which is related to CU calibration, directly impacts the accuracy of central dose measurements. Previous work by Renaud and Muir has demonstrated that dose response stability can be improved through diligent monitoring and by accounting for specific operational and environmental factors.^34^ Relative response constancy, relevant for ongoing symmetry measurements, may require the implementation of specialized EPID calibration protocols. For example, infrequent flood field calibration can help maintain consistency in EPID performance, which is essential when deploying EPID test suites for clinical QA.^35^, ^36^, ^37^ Emerging methods such as the Pixel Sensitivity Map (PSM) offer potential improvements along with the ability to obtain better absolute measurements.^38^, ^39^, ^40^ A PSM preserves the beam horns while removing only the EPID artifacts, rather than a typical flood field calibration which removes both the beam horns and EPID structural artifacts. A PSM approach is analogous to an array calibration procedure that would be performed on an ICP and would be routinely repeated, likely on an annual basis to control for relative response instability. This technique will be implemented in the future to enhance the accuracy of EPID images processed through the test suite.

The clinical significance of test validation results depends on how each institution implements these tests, making the interpretation of results the user's responsibility. Discrepancies identified in the focal spot test suite methodology, specifically underestimation in the radial direction and overestimation in the transverse direction, warrant further investigation, which will be addressed in future work. There may be multiple reasons for this discrepancy. The experiment was done on a Clinac with an AS500 EPID, which includes arm backscatter in the EPID images which may result in different results in the radial direction. Likewise, this test leverages the different heights between the jaws and the MLC in the treatment head, which are not finite devices therefore a specific point had to be chosen on the device to account for the height difference. Moreover, the differences in these distances are relatively small, which limits the sensitivity of this test. This is further confounded by the Y jaws and X jaws being at different heights which also changes the sensitivity of the radial and transverse measurements. Nonetheless, both the test suite EPID and ICP methods effectively guided beam steering back to the central axis of collimator rotation. Validation results can inform the establishment of clinical action thresholds; for instance, if the focal spot deviates by a specified amount in the test suite, it would trigger equipment investigation and realignment using an ICP. Similar to how detector systems used for daily QA monitoring may lead to output adjustments using a Farmer chamber, the EPID‐based test can monitor the focal spot and prompt adjustments using a more appropriate detector. Since formal recommendations for direct testing of focal spot are lacking, although discussed in AAPM Task Group Report 330 as being imperative for geometric accuracy and beam profile constancy, this test offers a practical checkpoint before and after repairs that may influence beam steering.^7^

Our analysis of focal spot and symmetry tests was limited to a single non‐clinical linac due to the beam steering required to introduce large discrepancies. This technique's limitation is that de‐tuning a machine is not practical within a clinical environment. By conducting this rigorous validation ourselves, we ensure readers do not need to repeat such extensive testing with asymmetry and focal spot values far beyond a typical clinical range. One solution is to conduct various QA commissioning measurements during system commissioning at the time of typical beam steering, thus establishing baseline values. Alternatively, simulations could generate artificial EPID images with various errors, enabling system testing without beam adjustments or compounding linac errors. These artificial images could be generated by inducing errors in commercial systems, such as Varian's (Palo Alto, CA) portal dosimetry algorithm, or by using free Monte Carlo packages like PRIMO.^41^ Alternative non‐computational methods for inducing errors include the insertion of objects (e.g., solid water) into the beam path to simulate beam deviations and the introduction of asymmetry through the use of half‐blocked fields.^42^, ^43^ Data have been preserved from this study and can be utilized in future iterations of the software, thereby eliminating the need to replicate the current study.

The test suite itself was created using a team science approach, engaging members of the Automated Quality Assurance Consortium from a variety of institutions with some grant support from Varian to The University of Michigan. This allowed us to leverage diverse experiences and account for different failure modes across multiple generations of Varian linacs. While the test suite is broadly applicable and can be implemented at any institution within the consortium, this study serves as a practical validation of the test suite itself. Validating EPID QA tests would be significantly enhanced through collaboration between end‐users and commercial software developers. While this study was limited to one vendor due to the funding source, Varian, theoretically the tests and methodology should be applicable to other linacs and vendors, with some modifications, as long as the vendor provides access to key data for their EPID for QA applications. In our work, we intentionally evaluate EPID results using software developed as part of the consortium rather than with the vendor QA system. If all vendors provided software tools for physicists to access key EPID data, regardless of the delivery system, then approaches such as use of a standard software suite would allow straightforward evaluation of linac performance. For this study, we created a testing space, or “sandbox,” for the test suite. The incorporation of a test environment for use during software commissioning is consistent with workflows to support safe implementation of the software, as outlined by Moran et al.^44^ This duplicate environment of the test suite database, analysis tools, and test suite, with certain safeguards removed, allowed us to introduce errors artificially without affecting the clinical QA database. This sandbox enabled repeated testing, data storage with annotations, and prevented database contamination. Providing users access to such a sandbox would also be advantageous when evaluating vendor‐supplied automated QA suites.

## 5 CONCLUSION

By comparing the AQA test suite's EPID analysis to traditional methods like the ICP and IC, we established a reliable framework for commissioning and integrating automated QA tools into clinical practice. This initial technical evaluation assessed the sensitivity of selected test suite analyses in detecting deviations introduced into a clinical EPID and linac system and was conducted in a single institution. Given the rapid development of EPID‐based QA tests and frequent updates to commercial image analysis packages, routine commissioning methodologies are essential for ensuring accurate QA results. Our findings highlight the importance of institutions customizing QA protocols to meet their specific needs while maintaining a comprehensive understanding of both automated and traditional methods. The sandbox environment enabled rigorous testing without compromising clinical data, illustrating the value of such innovations in developing and validating QA processes. As radiation therapy technologies evolve and become more complex, collaboration between end‐users and software developers will be crucial in refining and improving the efficiency of QA tools. Ultimately, adopting a balanced approach that combines efficient QA techniques with established methods will ensure optimal patient care and treatment accuracy in modern clinical settings.

## AUTHOR CONTRIBUTIONS

**Lana Critchfield**: Initial investigation; methodology; data collection; writing—original draft. **Cory Knill**: Initial investigation; methodology; data collection; writing—original draft. **Ellie Durussel**: Initial investigation; data collection. **Justin Mikell**: Initial investigation; methodology; data collection; test suite development; writing—review and editing. **Michael Barnes**: Initial investigation; methodology; interpretation of data; test suite development; writing—review and editing. **Michael Pillainayagam**: Data collection; writing—review and editing. **William Donahue**: Methodology; writing—review and editing. **Seng Boh Lim**: Methodology; writing—review and editing; test suite development; interpretation of data. **John DeMarco**: Methodology; writing—review and editing; test suite development. **Mario Perez**: Methodology; writing—review and editing; test suite development. **Nilendu Gupta**: Methodology; writing—review and editing; test suite development. **Hania Al‐Hallaq**: Methodology; writing—review and editing; test suite development. **Richard Popple**: Methodology; writing—review and editing; test suite development. **Jim Irrer**: initial investigation; methodology; test suite development. **Jean Moran**: Initial investigation; methodology; test suite development; writing—review and editing.

## CONFLICT OF INTEREST STATEMENT

The authors disclose that this study was supported in part by a grant from Varian.
